# Factors Associated with Early Rebleeding After Endoscopic Variceal Ligation in Cirrhotic Patients: A Retrospective Cohort Study

**DOI:** 10.3390/jcm15062372

**Published:** 2026-03-20

**Authors:** Simona Juncu, Ana-Maria Sîngeap, Horia Minea, Andreea Lungu, Alexandru Sebastian Cotleț, Ana-Maria Buzuleac, Raluca Avram, Cristina Muzica, Laura Huiban, Irina Gîrleanu, Alina Ecaterina Jucan, Georgiana-Emmanuela Gîlcă-Blanariu, Andrei Ciobica, Alin Ciobica, Anca Trifan, Camelia Cojocariu

**Affiliations:** 1 Department of Gastroenterology, Faculty of Medicine, “Grigore T. Popa” University of Medicine and Pharmacy, 700115 Iasi, Romania; juncu_simona-stefania@d.umfiasi.ro (S.J.); horia-octav.minea@umfiasi.ro (H.M.); zvincu.andreea@d.umfiasi.ro (A.L.); alexandru-sebastian.cotlet@d.umfiasi.ro (A.S.C.); buzuleac.ana-maria@d.umfiasi.ro (A.-M.B.); avram_raluca-ioana@d.umfiasi.ro (R.A.); cristina.muzica@umfiasi.ro (C.M.); laura.huiban@umfiasi.ro (L.H.); irina.girleanu@umfiasi.ro (I.G.); ghiata.alina-ecaterina@d.umfiasi.ro (A.E.J.); georgiana-emmanuela.gilca@umfiasi.ro (G.-E.G.-B.); anca.trifan@umfiasi.ro (A.T.); cojocariu.salloum@umfiasi.ro (C.C.); 2 Institute of Gastroenterology and Hepatology, “St. Spiridon” University Hospital, 700111 Iasi, Romania; 3 CENEMED Platform for Interdisciplinary Research, University of Medicine and Pharmacy “Grigore T. Popa”, 700115 Iasi, Romania; alin.ciobica@uaic.ro; 4 Department of Physiology, “Grigore T. Popa” University of Medicine and Pharmacy, 16 Universitatii Street, 700115 Iasi, Romania; andrei.ciobica@yahoo.com; 5 Department of Biology, Faculty of Biology, “Alexandru Ioan Cuza” University of Iasi, Bd. Carol I No. 20A, 700505 Iasi, Romania; 6 “Ioan Haulica” Institute, Apollonia University, 11 Păcurari Street, 700511 Iasi, Romania; 7 Academy of Romanian Scientists, 3 Ilfov, 050044 Bucharest, Romania

**Keywords:** cirrhosis, portal hypertension, esophageal varices, endoscopic variceal ligation, early rebleeding, post-banding ulcer, predictors, mortality

## Abstract

**Background:** Early rebleeding after endoscopic variceal ligation (EVL) represents a serious complication in patients with cirrhosis and is associated with poor short-term outcomes. This study aimed to identify independent predictors of early rebleeding after EVL, with a particular focus on distinguishing factors associated with variceal rebleeding from those related to post-banding ulcer (PBU) bleeding, and to assess predictors of six-week mortality. **Methods**: We conducted a retrospective cohort study including 217 cirrhotic patients who underwent first emergency EVL for an index episode of esophageal variceal bleeding at a tertiary referral center. Early rebleeding was defined as recurrent upper gastrointestinal bleeding occurring between days 6 and 42 after the index EVL. **Results**: Early rebleeding occurred in 38/217 patients (17.5%): 27/38 (71.1%) variceal rebleeding and 11/38 (28.9%) PBU rebleeding. In multivariable logistic regression analysis, lower hemoglobin (OR = 0.19, 95% CI: 0.067–0.539, *p* = 0.002) and a higher albumin–bilirubin (ALBI) grade (OR = 24.94, 95% CI: 1.134–548.342, *p* = 0.041) were independently associated with increased odds of early variceal rebleeding, whereas a higher number of bands applied during index EVL (OR = 0.52, 95% CI: 0.302–0.896, *p* = 0.019) was independently associated with reduced odds of rebleeding, with excellent model discrimination (area under the curve [AUC] 0.981; 95% CI: 0.959–1.000). For PBU rebleeding, lower fibrinogen level was the only independent predictor (OR = 0.957, 95% CI: 0.916–1.000, *p* = 0.047), with strong discriminative performance (AUC 0.945; 95% CI: 0.909–0.982). Model for End-Stage Liver Disease (MELD) score, serum albumin, platelet count, and PBU rebleeding independently predicted six-week mortality. **Conclusions:** Markers of liver function, along with endoscopic parameters, predict early rebleeding after EVL, emphasizing the importance of the complete assessment of cirrhotic patients for refined risk stratification and tailored post-EVL management.

## 1. Introduction

Variceal bleeding (VB) is one of the most dramatic emergencies in gastroenterology and remains a leading cause of death in patients with liver cirrhosis. Approximately 52% of cirrhotic patients develop gastrointestinal varices, with a prevalence of 50–60% in compensated cirrhosis and up to 85% in decompensated disease [1]. The standard management of acute variceal bleeding includes hemodynamic resuscitation, prophylactic antibiotics, vasoactive agents (e.g., terlipressin, somatostatin, and their analogues), and endoscopic intervention. The implementation of these combined therapeutic strategies has significantly reduced mortality over recent decades, from nearly 50% to approximately 10–20% [2].

Endoscopic variceal ligation (EVL), either alone or in combination with nonselective beta-blockers (NSBBs), plays a pivotal role in both primary and secondary prophylaxis in patients with high-risk esophageal varices, effectively reducing bleeding incidence and associated complications [3]. Despite these advances, early rebleeding after EVL remains a frequent and severe complication, occurring in up to 30–40% of patients, and is linked to increased morbidity and mortality [4,5].

The hepatic venous pressure gradient (HVPG) is the gold-standard hemodynamic parameter for assessing portal hypertension. HVPG values exceeding 10 mmHg are associated with variceal development, while values above 12 mmHg accurately predict bleeding risk [6]. An HVPG ≥ 20 mmHg has been identified as the strongest predictor of poor prognosis in variceal bleeding, as highlighted in European Society of Gastrointestinal Endoscopy (ESGE) guidelines. However, due to its limited availability in routine clinical practice, surrogate clinical tools such as the Child–Pugh and MELD scores are more commonly used for risk stratification [7].

According to the Baveno VII consensus, five-day treatment failure in variceal bleeding is defined as the inability to achieve initial hemostasis or the occurrence of rebleeding within the first five days following the index intervention [8]. In addition, a recent review clearly distinguishes between treatment failure, defined as rebleeding within the first five days, and failure of secondary prophylaxis, referring to rebleeding occurring after five days [2]. The American guidelines further emphasize that the highest risk period for rebleeding extends through the first six weeks following EVL, most commonly because of persistent varices or endoscopic ligation-associated ulcers [9].

Definitions of early rebleeding vary among cohort studies evaluating its associated risk factors. A recent meta-analysis including 16 studies reported an early rebleeding rate of 10.5% within the first six weeks after EVL, with 5.7% of cases attributed to post-banding ulcer (PBU) bleeding. Identified risk factors include the presence of gastric varices, a higher number of bands applied, peptic esophagitis, proton pump inhibitor (PPI) use, hepatocellular carcinoma (HCC), low hemoglobin levels, elevated MELD scores, advanced age, emergency EVL, large varices, high-risk endoscopic stigmata, elevated bilirubin, hypoalbuminemia, and Child–Pugh class C cirrhosis [5].

Identifying these risk factors may contribute to the development of predictive models which could stratify high-risk patients, allowing an earlier intervention and more tailored management strategies, aiming to prevent further hepatic decompensation. In this context, this study aimed to assess independent predictive factors of early rebleeding after EVL, with a particular focus on distinguishing predictors of variceal rebleeding from those associated with PBU bleeding. Improved awareness of these factors may facilitate better identification of high-risk individuals and ultimately improve clinical outcomes in cirrhotic patients.

## 2. Materials and Methods

### 2.1. Patients

**Figure 1:**
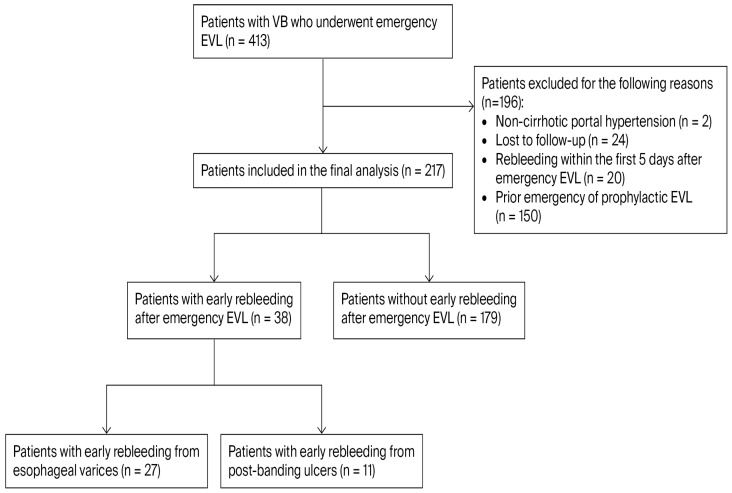
Flowchart of patient selection and study population.

We retrospectively evaluated 217 consecutive adult patients presenting with VB who underwent emergency upper gastrointestinal endoscopy (UGE) between January 2019 and April 2025 at the Institute of Gastroenterology and Hepatology, Iași, Romania, a tertiary referral center. Eligible participants were adults (≥18 years) with a diagnosis of liver cirrhosis, established based on clinical features, laboratory findings, and imaging studies, who presented with a first episode of acute variceal bleeding and underwent emergency EVL. All included patients had provided informed consent for UGE and EVL at the time of the index hospitalization. Exclusion criteria comprised: lack of informed consent; absence of UGE confirmation of the bleeding source at rebleeding; non-cirrhotic portal hypertension; loss to follow-up within the six-week observation period; prior episodes of variceal hemorrhage; previous emergency or prophylactic EVL; and rebleeding occurring within the first five days after the index bleeding episode despite initial endoscopic hemostasis (defined as treatment failure). The patient selection process is illustrated in Figure 1.

Baseline assessments at admission comprised demographic data, clinical presentation of upper gastrointestinal bleeding, routine laboratory parameters, abdominal ultrasound parameters of portal hypertension, liver function assessment using validated clinical scores, comorbidities, concomitant treatments, compliance with scheduled consolidation EVL sessions, and endoscopic characteristics. The primary objective of the study was to identify predictive factors of early rebleeding, defined as recurrent bleeding occurring between days 6 and 42 after the index emergency EVL, with separate analyses for variceal rebleeding and PBU bleeding. The secondary objective was to identify predictors of six-weeks mortality.

The study was conducted in accordance with the ethical principles of the Declaration of Helsinki and was approved by the Institutional Ethics Committee. Given the retrospective design, use of anonymized data, and the fact that all procedures and laboratory tests were performed as part of routine clinical care, the requirement for individual informed consent for study participation was waived.

### 2.2. Initial Management and Endoscopic Procedure

Upper gastrointestinal endoscopy was performed within 12 h of hospital presentation in all patients, following hemodynamic stabilization. Vasoactive agents were initiated immediately upon emergency admission in all patients with suspected variceal bleeding. Antiplatelet and anticoagulant therapies were managed according to current European Society of Gastrointestinal Endoscopy (ESGE) guidelines, ensuring standardized pre- and post-endoscopic care.

Endoscopic procedures were performed using an Olympus endoscope (Olympus Corporation, Tokyo, Japan) with a standard 9 mm diameter, equipped with a Speedband Superview Super 7™ multiple band ligator system (Boston Scientific Corporation, Marlborough, MA, USA). Esophageal varices were classified according to contemporary guideline-based criteria as small varices with red signs (<5 mm diameter) and large varices with red signs (>5 mm diameter) [8]. During EVL, target varices were endoscopically identified and then suctioned into the ligator chamber. Adequate suction was confirmed by reddening of the endoscopic field, after which the elastic band was released by clockwise rotation of the deployment handle. Bands were subsequently applied in an ascending pathway along the esophagus. The total number of bands applied during the index procedure was recorded for each patient.

### 2.3. Follow-Up and Outcome Definitions

Following EVL, patients received vasoactive therapy (terlipressin or somatostatin) for up to five days, in accordance with international guidelines. Pre-endoscopy PPIs were stopped immediately after confirmation of VB and were continued only in cases of PBU. All patients received antibiotic prophylaxis with ceftriaxone 1 g/day (or an alternative regimen in case of allergy) for up to seven days, as well lactulose for the prevention of hepatic encephalopathy. Oral intake was resumed 24 h after the procedure. After discontinuation of vasoactive therapy, non-selective beta-blockers (NSBBs) were initiated for secondary prophylaxis of VB. Follow-up EVL sessions were scheduled every 2–4 weeks, in accordance with guidelines recommendations. Early rebleeding after EVL was defined as the recurrence of hematemesis and/or melena between days 6 and 42 after the index emergency EVL, after initial successful endoscopic hemostasis, accompanied by at least one of the following: a decrease in hemoglobin ≥ 20 g/L, the requirement for transfusion of more than two units of packed red blood cells, or the occurrence of hypovolemic shock after initial endoscopic treatment, in accordance with the Baveno VII consensus [8]. PBU bleeding was defined as early rebleeding following initial successful hemostasis, with endoscopic confirmation of bleeding from a PBU in the absence of active variceal bleeding and meeting the same clinical and severity criteria as defined for early bleeding.

All rebleeding episodes were managed according to the standard-of-care algorithm for acute VB, including repeat endoscopic therapy and escalation to rescue treatments when clinically indicated.

### 2.4. Statistical Analysis

Continuous variables were analyzed using one-way analysis of variance (ANOVA), with Fisher’s least significant difference (LSD) post hoc tests applied for pairwise comparisons when overall significance was observed (*p* < 0.05). Post hoc analyses compared three predefined groups: (1) no rebleeding, (2) variceal rebleeding, and (3) PBU bleeding. Categorical variables were assessed using the chi-square test of independence, with two predefined pairwise comparisons: (a) no rebleeding versus variceal rebleeding and (b) no rebleeding versus PBU bleeding. Following the univariate analysis, we performed a multivariable analysis, with the aim to identify the independent predictors of early variceal rebleeding and from PBU. The multivariable logistic regression comprised all significant variables at the univariate analysis. The discriminative ability of the final logistic regression model was evaluated using a Receiver Operating Characteristic (ROC) curve analysis and a Kaplan–Meier (KM) survival analysis was conducted to estimate the time to rebleeding events. Furthermore, a binary logistic regression analysis was conducted to evaluate the predictors of mortality rate among the patients included in the study. Statistical analyses were performed using SPSS version 17.0 (IBM Corp., Chicago, IL, USA) and a *p*-value < 0.05 was considered a marker of statistical significance.

## 3. Results

### 3.1. Study Population and Baseline Characteristics

According to the predefined inclusion and exclusion criteria, a total of 217 patients were included in this retrospective cohort study: 153 male (70.5%) and 64 female patients (29.5%). The etiologies of liver cirrhosis were alcohol-related in 144 cases (66.4%), viral hepatitis in 40 cases (18.4%), mixed alcohol- and virus-related etiology in 26 cases (12%), and other causes, including autoimmune liver diseases, metabolic-associated steatotic liver disease, and haemochromatosis in 7 cases (3.2%).

### 3.2. Incidence and Timing of Early Rebleeding

Patients were divided into two groups: those with early rebleeding (38 patients; 17.5%) and those without early rebleeding (179 patients; 82.5%). Within the early rebleeding group, 27 patients (71.1%) experienced variceal rebleeding, while 11 patients (28.9%) developed PBU bleeding (Figure 1). The mean time to early rebleeding was 18.5 ± 10.1 days in patients with variceal rebleeding and 17.8 ± 7.4 days in those with PBU bleeding.

### 3.3. Early Rebleeding and Six-Week Mortality

Among patients with early variceal rebleeding, the mortality rate was 11.1% (3/27). In contrast, patients with PBU rebleeding exhibited a higher mortality rate of 36.4% (4/11). In the non-rebleeding group (n = 179), 13 deaths were recorded during follow-up, corresponding to a mortality rate of 7.3%.

### 3.4. Comparison of Baseline and Endoscopic Characteristics According to Rebleeding Status

**Figure 2:**
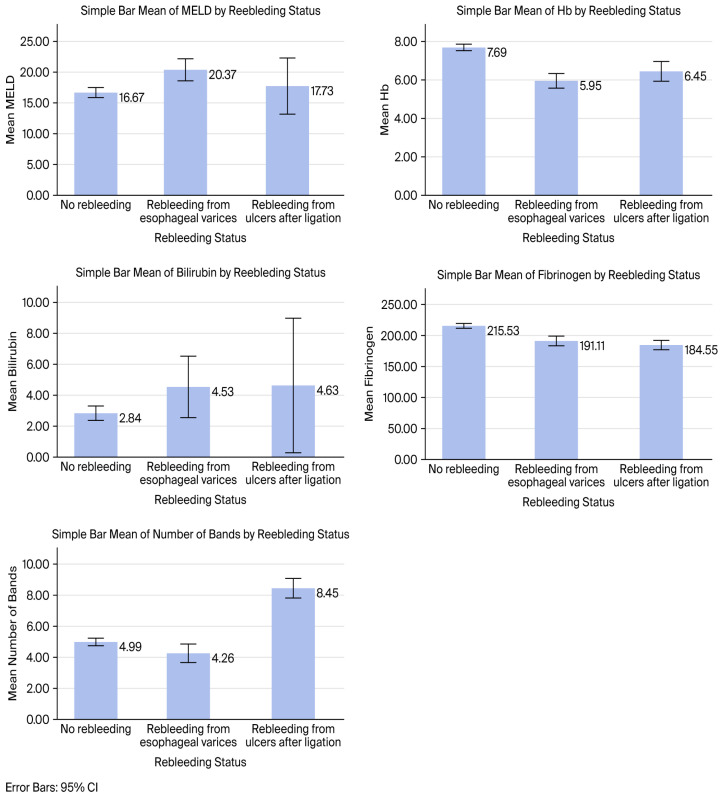
Comparison of MELD score, hemoglobin, total bilirubin, fibrinogen, and number of bands across rebleeding groups (no rebleeding, variceal rebleeding, and post-banding ulcer bleeding). Data are presented as mean values with error bars representing 95% confidence intervals.

Baseline clinical, laboratory, ultrasound, and endoscopic characteristics, including hepatic encephalopathy (HE), spontaneous bacterial peritonitis (SBP), acute kidney injury (AKI), acute-on-chronic liver failure (ACLF), and portal vein thrombosis (PVT), were compared across the three predefined groups (no rebleeding, early variceal rebleeding, and PBU bleeding) (Table 1 and Table 2). Mean values of MELD score, hemoglobin, total bilirubin, fibrinogen, and number of bands across the three rebleeding groups are illustrated in Figure 2.

Continuous variables were compared using one-way ANOVA test (Table 3), followed by Fisher’s LSD post hoc testing when overall significance was observed (Table 4).

One-way ANOVA test revealed significant differences among the three rebleeding status groups (no rebleeding, variceal rebleeding, and PBU rebleeding) for all five continuous variables: MELD score, hemoglobin, total bilirubin, fibrinogen, and number of bands. Following ANOVA test, pair-wise post hoc LSD comparisons were performed to identify specific between-group differences. Post hoc analysis showed that MELD score, total bilirubin, and hemoglobin differed significantly between the non-rebleeding and variceal rebleeding groups, while fibrinogen levels were higher in non-rebleeding patients compared to both rebleeding groups. Regarding the number of bands, significant differences were observed among the three groups, with the highest values in rebleeding patients and a significant difference between the variceal and post-banding ulcer rebleeding groups.

### 3.5. Factors Associated with Early Variceal Rebleeding

For categorical variables, associations with early variceal rebleeding were assessed using the Chi-square test. The results of the univariate Chi-square analysis exploring clinical and endoscopic factors associated with the early variceal rebleeding are summarized in Table 5.

**Figure 3:**
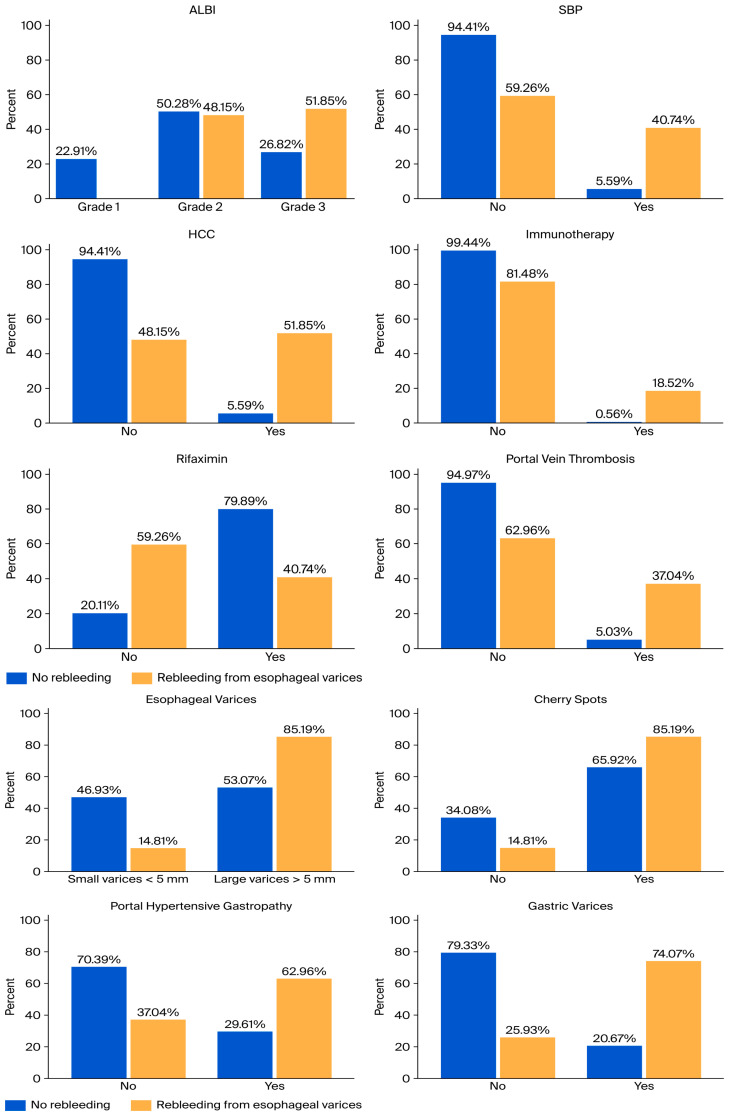
Distribution of clinically relevant variables according to variceal rebleeding status. Bar plots illustrate the percentage distribution of patients with and without early variceal rebleeding for variables showing statistically significant associations in the univariate Chi-square analysis, including ALBI grade, spontaneous bacterial peritonitis, hepatocellular carcinoma, immunotherapy, rifaximin use, portal vein thrombosis, esophageal variceal size, cherry spots, portal hypertensive gastropathy, and gastric varices.

Several variables showed statistically significant associations with early variceal rebleeding in the univariate analysis. These included SBP (χ^2^ = 31.670, *p* < 0.001, V = 0.392), HCC (χ^2^ = 48.788, *p* < 0.001, V = 0.487), immunotherapy (χ^2^ = 26.761, *p* < 0.001, V = 0.360), rifaximin use (χ^2^ = 19.053, *p* < 0.001, V = 0.304), portal vein thrombosis (χ^2^ = 28.710, *p* < 0.001, V = 0.373), esophageal varices size (χ^2^ = 9.887, *p* = 0.002, V = 0.219), cherry spots (χ^2^ = 4.031, *p* = 0.045, V = 0.14), portal hypertensive gastropathy (χ^2^ = 11.634, *p* = 0.001, V = 0.238), and gastric varices (χ^2^ = 33.432, *p* < 0.001, V = 0.403). Effect size estimation using Cramér’s V indicated moderate to moderate-strong associations for SBP, HCC, immunotherapy, rifaximin use, PVT, and gastric varices, while small to moderate associations were observed for esophageal varices (EV) size, portal hypertensive gastropathy, and cherry spots. No statistically significant associations were identified for ACLF, AKI, HE, ascites, active bleeding at index endoscopy, consolidation banding sessions (all *p* > 0.05). These findings are illustrated in Figure 3 which present clustered bar plots showing the percentage distribution of patients with and without early variceal rebleeding for variables that showed statistically significant associations in the univariate Chi-square analysis.

Following the univariate analysis—one-way ANOVA (with Fisher’s LSD post hoc comparisons) for continuous variables and the Chi-square test for categorical variables, a multivariable logistic regression analysis was performed to identify independent predictors of early variceal rebleeding. The full logistic regression model including all selected predictors was statistically significant, χ^2^ (16, N = 217) = 123.685, *p* < 0.001, indicating a robust ability to distinguish between patients who experienced early variceal rebleeding and those who did not. In addition, the Hosmer–Lemeshow goodness-of-fit test was conducted to evaluate how well the model fitted the observed data. The results indicated an excellent model fit, χ^2^ (8) = 7.04, *p* = 0.532), suggesting no significant difference between observed and predicted outcomes. The model demonstrated substantial explanatory power, with a Nagelkerke R Square of 0.822, indicating that the predictors included in the model explained a high proportion of the variance in rebleeding status.

Additionally, the model showed excellent predictive performance, with an overall classification accuracy of 98.2%. It correctly identified 99.5% of patients without early rebleeding and 88.9% of those with early variceal rebleeding.

Among continuous variables, hemoglobin level and the number of bands applied during the index EVL emerged as independent predictors of early variceal rebleeding. Each unit increase in hemoglobin level was associated with a significant lower risk of rebleeding (OR = 0.19, 95% CI: 0.067–0.539, *p* = 0.002). Similarly, a higher number of bands applied during the index procedure was associated with reduced odds of rebleeding (OR = 0.52, 95% CI: 0.302–0.896, *p* = 0.019). Among categorical variables, ALBI grade was the only independent predictor of early variceal rebleeding (OR = 24.94, 95% CI: 1.134–548.342, *p* = 0.041).

Variables included in the multivariable logistic regression models were selected based on statistical significance in univariate analysis (*p* < 0.05) and clinical relevance. To avoid multicollinearity, composite liver severity scores (MELD, ALBI, Child–Pugh) were not entered simultaneously with their individual components in the same model. Multicollinearity was assessed using variance inflation factors (VIFs), and no significant collinearity was identified.

**Figure 4:**
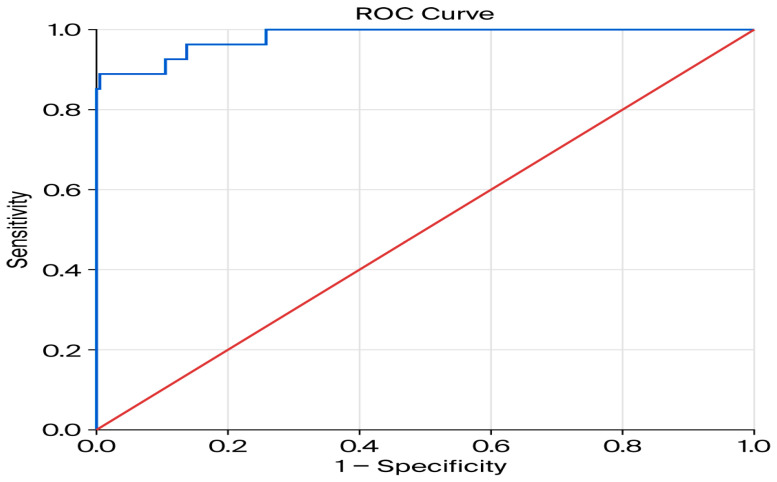
Receiver operating characteristic (ROC) curve showing the discriminative ability of the multivariable logistic regression model for early variceal rebleeding (AUC = 0.981, 95% CI: 0.959–1.000).

The discriminative ability of the final logistic regression model was evaluated using a Receiver Operating Characteristic (ROC) curve analysis. The model demonstrated excellent predictive performance for early variceal rebleeding, with an Area Under the Curve (AUC) of 0.981 (95% CI: 0.959 to 1.000) (Figure 4). This result was highly statistically significant (*p* < 0.001), indicating an outstanding ability of the model to discriminate between patients who will and will not experience early variceal rebleeding. An AUC value of 0.981 corresponds to a 98.1% probability that the model will correctly rank a patient who experiences early variceal rebleeding higher than a patient who does not. Given the relatively small number of rebleeding events and the potential risk of overfitting, internal validation of the multivariable model was performed using bootstrap resampling (1000 iterations). The bootstrap analysis confirmed the stability of hemoglobin level and number of bands as predictors of early variceal rebleeding, whereas the association for ALBI grade showed greater variability across resampled datasets.

### 3.6. Factors Associated with Post-Banding Ulcer Rebleeding

A series of Chi-square tests of independence were also performed to explore association between clinical and endoscopic factors and the risk of early PBU rebleeding. The results of this univariate analysis are summarized in Table 6.

**Figure 5:**
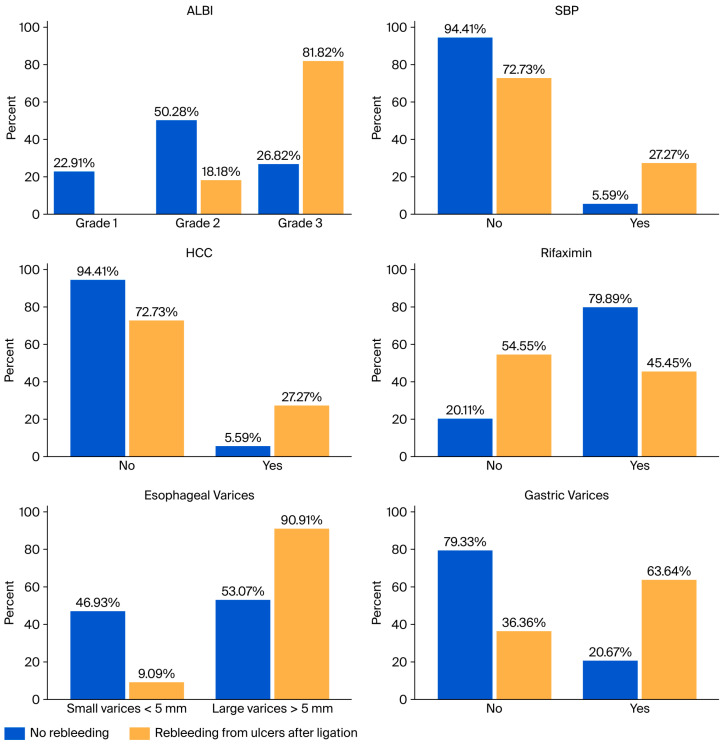
Distribution of clinical and endoscopic variables according to post-banding ulcer rebleeding status. Bar plots illustrate the percentage distribution of patients with and without post-banding ulcer rebleeding for variables showing statistically significant associations in the chi-square analysis, including ALBI grade, spontaneous bacterial peritonitis, hepatocellular carcinoma, rifaximin use, esophageal variceal size, and gastric varices.

The Chi-square analysis identified several variables significantly associated with early PBU rebleeding. Higher ALBI grades (χ^2^ = 15.175, *p* = 0.001, Cramér’s V = 0.283), the presence of SBP (χ^2^ = 7.646, *p* = 0.006, V = 0.201), HCC (χ^2^ = 7.646, *p* = 0.006, V = 0.201), and Rifaximin use (χ^2^ = 7.136, *p* = 0.008, V = 0.194) were significantly associated with PBU rebleeding. In addition, large EV (χ^2^ = 6.001, *p* = 0.014, V = 0.178) and the presence of gastric varices (χ^2^ = 10.751, *p* = 0.001, V = 0.238) were associated with an increased risk of rebleeding. No statistically significant associations (*p* > 0.05) were identified for gender, etiology of cirrhosis, clinical presentation, ACLF, AKI, immunotherapy, HE, ascites, PVT, active hemorrhage, cherry spots, portal hypertensive gastropathy, and consolidation sessions. Six-week mortality was also significantly associated with PBU rebleeding (χ^2^ = 10.773, *p* = 0.001, V = 0.238). These findings are illustrated in Figure 5, which presents the percentage distribution of patients with and without post-banding ulcer rebleeding across the significant variables.

Following the univariate analysis, a binary logistic regression analysis was performed to identify independent predictors of early PBU rebleeding. The full logistic regression model was statistically significant, χ^2^ (12, N = 217) = 35.347, *p* < 0.001, indicating an overall ability to distinguish between patients with and without PBU rebleeding. The model demonstrated moderate explanatory power (Nagelkerke R Square = 0.455). Furthermore, the Hosmer–Lemeshow goodness-of-fit test indicated an excellent fit (χ^2^ (8) = 3.23, *p* = 0.919). The overall classification accuracy was 95.4%; however, while the model correctly classified 99% of patients without rebleeding, sensitivity for predicting PBU rebleeding was low (27.3%).

Among continuous variables, fibrinogen was the only independent predictor of early PBU. Each unit increase in fibrinogen level was associated with a significantly lower risk of rebleeding (OR = 0.957, 95% CI: 0.916–1.000, *p* = 0.047). None of the categorical variables retained statistical significance in the multivariable analysis.

**Figure 6:**
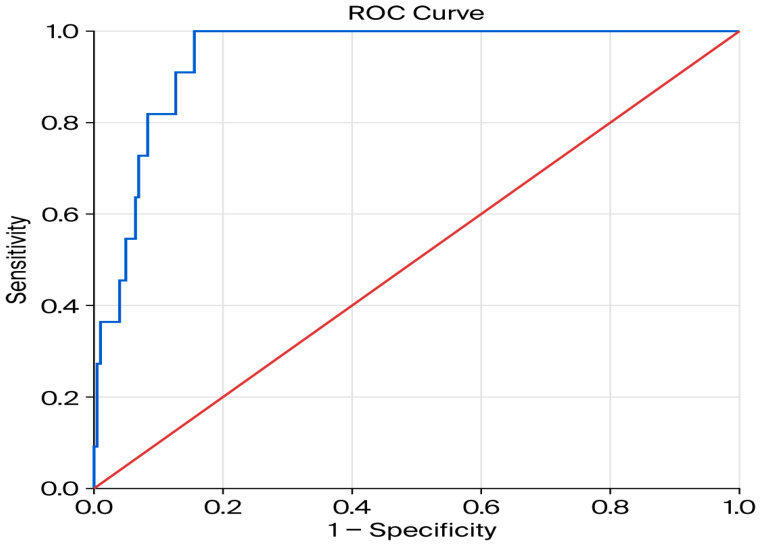
Receiver operating characteristic (ROC) curve showing discriminative ability of the multivariable logistic regression model for post-banding ulcer rebleeding (AUC = 0.945, 95% CI: 0.909–0.982).

The discriminative ability of the logistic regression model was also evaluated using ROC curve analysis. The model demonstrated very strong discriminative performance for early PBU rebleeding, with AUC of 0.945 (95% CI: 0.909 to 0.982) (Figure 6). This result was highly statistically significant (*p* < 0.001), indicating an excellent ability of the model to distinguish between patients who will and will not experience early PBU rebleeding. This AUC value corresponds to a 94.5% probability that the model will correctly rank a patient who experiences PBU rebleeding higher than a patient who does not.

### 3.7. Rebleeding-Free Survival After EVL

**Figure 7:**
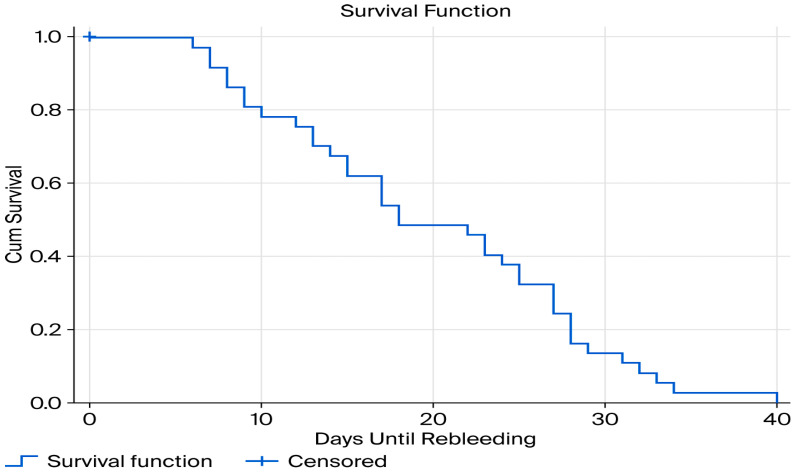
Kaplan–Meier curve for rebleeding-free survival following emergency endoscopic variceal ligation. The curve depicts time to first early rebleeding after successful endoscopic hemostasis. The median rebleeding-free survival time was 18 days (95% CI: 12–24 days).

A Kaplan–Meier survival analysis was conducted to estimate rebleeding-free survival following emergency EVL. The median rebleeding-free survival was 18 days (95% CI: 12–24 days) (Figure 7). The mean time until rebleeding was 19.9 days (95% CI: 17–22.8 days).

### 3.8. Factors Associated with Six-Week Mortality

**Figure 8:**
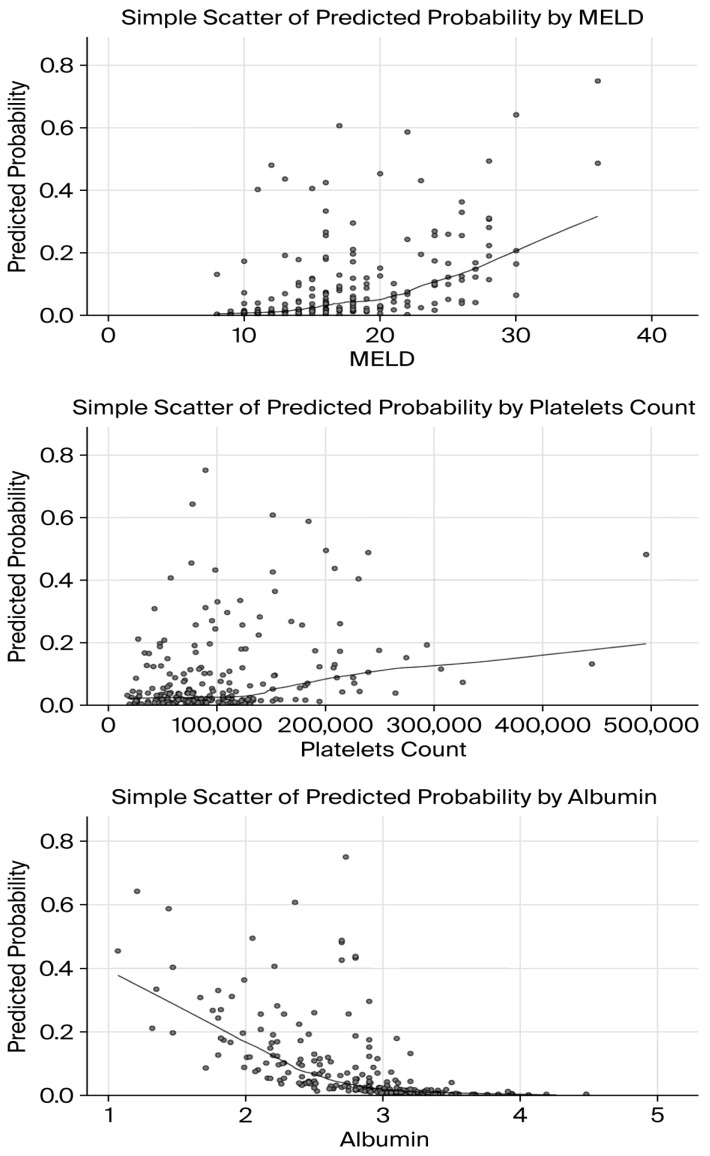
Predicted probability of six-week mortality according to MELD score, platelet count, and serum albumin. Scatter plots illustrate the association between MELD score, platelet count, and serum albumin levels and the predicted probability of six-week mortality as estimated by the multivariable logistic regression model.

**Figure 9:**
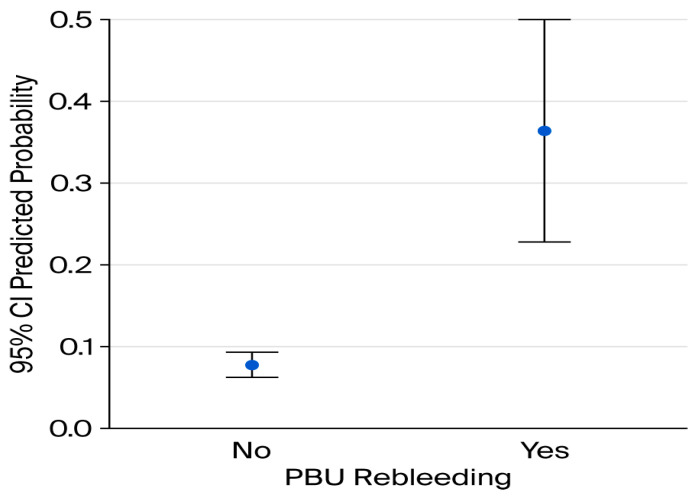
Predicted probability of six-week mortality according to post-banding ulcer rebleeding status. Bars represent the mean predicted probability of six-week mortality estimated by the multivariable logistic regression model, stratified by the presence or absence of post-banding ulcer rebleeding. Error bars indicate 95% confidence intervals.

A binary logistic regression analysis was performed to identify independent predictors of six-week mortality among patients included in the study. The model incorporating MELD score, platelet count, PBU rebleeding, and serum albumin level was statistically significant (χ^2^ (4, N = 217) = 34.86, *p* < 0.001), explaining 32.3% of the variance in mortality (Nagelkerke R^2^ = 0.323). Higher MELD scores (OR = 1.11, *p* = 0.024) and platelet counts (OR = 1.001, *p* = 0.001) were independently associated with an increased risk of six-week mortality, while higher serum albumin levels had a protective effect (OR = 0.16, *p* = 0.001) (Figure 8). PBU emerged as the strongest predictor of six-week mortality, markedly increasing the odds of death (OR = 13.7, *p* = 0.001) (Figure 9).

## 4. Discussion

Early rebleeding following endoscopic treatment of variceal bleeding remains a major determinant of short-term prognosis in patients with liver cirrhosis. Despite substantial advances in the management of acute variceal hemorrhage, mortality after a first bleeding episode continues to range between 15% and 25%, largely driven by advanced liver dysfunction, associated comorbidities, and the occurrence of early rebleeding events [10]. Previous studies have reported early rebleeding rates after EVL ranging from 4.8% to 15.6% [1], with some cohorts describing rates as high as 20.8% [11], underscoring the clinical relevance of this complication. Consistent with these data, early rebleeding occurred in 17.5% of patients in our cohort, confirming that early post-EVL hemorrhage remains a frequent and clinically meaningful event in real-world practice.

Importantly, early rebleeding is not a uniform entity. When it occurs, it is often severe, difficult to control, and associated with high mortality rates, reported to range between 26.9% and 38.3% [1], substantially exceeding mortality observed after the index bleeding episode. However, most available studies have analyzed early rebleeding as a single outcome, without differentiating between variceal rebleeding and PBU bleeding, despite their distinct pathophysiological mechanisms and potentially different prognostic implications. In this context, the present study provides a more granular evaluation of early rebleeding after EVL by separately analyzing predictors and outcomes associated with variceal rebleeding and PBU bleeding, thereby addressing an important gap in the existing literature.

In our study, according to the Kaplan–Meier survival analysis, the median time to early rebleeding was 18 days (95% CI: 12–24), with a mean time to rebleeding of 19.9 days (95% CI: 17–22.8). These findings are consistent with subgroup analyses, in which the mean time to rebleeding was 18.5 ± 10.1 days for variceal rebleeding and 17.8 ± 7.4 days for PBU bleeding. The close concordance between Kaplan–Meier estimates and subgroup analyses suggests relatively homogeneous temporal pattern of rebleeding across etiologies, highlighting a critical vulnerability window during the second and third weeks following index EVL. While some studies have reported shorter intervals to rebleeding (9.3 ± 3.5 days), these observations were often derived from cohorts with shorter follow-up periods, typically limited to 14 days [12], which may underestimate later rebleeding events captured in our six-week observation window.

Post-banding ulcer bleeding represents a distinct and clinically relevant cause of early rebleeding after EVL, with important prognostic implications. In our cohort, PBU bleeding accounted for 5.1% of all patients and 28.9% of early rebleeding events, a proportion consistent with previously reported incidences ranging between 4.6% and 9.2% [13,14,15]. However, more recent studies have described substantially higher rates of PBU bleeding, reaching up to 17.5% [16], likely reflecting the increasing use of EVL and improved survival after index variceal bleeding, which exposes patients to later complications of endoscopic therapy. These data suggest that PBU bleeding is not a rare event and may be increasingly encountered in contemporary clinical practice.

Beyond its incidence, PBU bleeding carries a disproportionate impact on short-term mortality. In our study, patients who developed early PBU rebleeding exhibited a six-week mortality rate of 36.4%, markedly higher than that observed in patients with variceal rebleeding (11.1%) and in those without early rebleeding (7.3%). This finding aligns with previous reports describing mortality rates between 23.8% [12] and 63.4% [17] among patients with PBU bleeding and underscores the particularly poor prognosis associated with this complication. Importantly, these observations reinforce the concept that PBU bleeding should not be regarded as a minor or purely local adverse event of EVL, but rather as a marker of advanced disease severity and biological vulnerability in cirrhotic patients.

In contrast to early PBU rebleeding, early variceal rebleeding appears to be more closely linked to the severity of underlying liver dysfunction. Previous studies have consistently demonstrated the prognostic value of liver disease severity scores, including MELD-Na [18], Child–Pugh [4], and platelet–albumin–bilirubin (PALBI) [19], in predicting early rebleeding after EVL. Our results are consistent with previously published data, as ALBI grade, a validated liver function assessment tool, emerged as an independent predictor of early variceal rebleeding (OR = 24.94, 95% CI: 1.134–548.342, *p* = 0.041), underscoring the central role of hepatic functional reserve in determining post-hemostasis stability.

Among endoscopic and bleeding-related parameters, both hemoglobin level at admission and the number of bands applied during the index EVL independently predicted early variceal rebleeding in our cohort. Higher hemoglobin levels were associated with a significantly lower risk of rebleeding, likely reflecting less severe initial hemorrhage, improved hemodynamic stability, and a more favorable systemic milieu for clot formation and mucosal healing. This observation is consistent with previous reports identifying low admission hemoglobin as a marker of increased early rebleeding risk following EVL [5,20].

The relationship between the number of bands applied during EVL and the risk of rebleeding remains controversial in the literature. While some studies have suggested that a higher number of bands may increase the risk of early rebleeding, possibly by enlarging mucosal injury and ulcer burden [21], insufficient ligation during the index procedure may leave residual high-risk varices untreated. In this regard, Jung et al. showed that minimal EVL, targeting only actively bleeding varices, was associated with higher early rebleeding rates compared with more extensive ligation strategies [22]. Moreover, a recent meta-analysis including 16 prospective and retrospective studies reported that a higher number of bands applied during endoscopy and lower hemoglobin levels at admission were associated with an increased risk of early rebleeding after EVL [5]. However, that analysis did not distinguish between variceal rebleeding and post-banding ulcer bleeding, which may account for the apparent discrepancy with our findings.

In our study, a higher number of bands applied during the index procedure was independently associated with a reduced risk of early variceal rebleeding. This finding supports the concept that more complete eradication of high-risk variceal segments during the initial session may outweigh the potential risks associated with additional mucosal trauma, at least with respect to variceal rebleeding.

The risk of rebleeding after EVL appears to be influenced by coagulopathy, as several studies have identified prothrombin time and INR as independent predictors of early rebleeding [23,24]. In our cohort, fibrinogen emerged as an independent predictor of early PBU rebleeding, with each unit increase in fibrinogen level being associated with a significantly reduced risk of rebleeding (OR = 0.957, *p* = 0.047). Similar findings were reported by Giannini et al. [25], who showed that patients with rebleeding had significantly lower fibrinogen levels compared with non-bleeders (146 mg/dL vs. 230 mg/dL, *p* = 0.009).

Current international guidelines report a six-week mortality rate of 10–20% among patients with acute VB [26], which is similar with that reported in our study among patients with variceal rebleeding (11.1%). In contrast, patients who developed early PBU rebleeding exhibited a marked higher six-week mortality rate (36.4%), substantially exceeding that observed in patients without early rebleeding (7.3%). This difference was more pronounced than that reported in other studies, where mortality rates were 30.8% in rebleeding patients and 21.8% in non-rebleeding patients [27]. In our study, MELD score, serum albumin level, platelet count, and the presence of PBU independently predicted six-week mortality. Among these factors, PBU rebleeding emerged as the strongest predictor of mortality, while higher serum albumin levels demonstrated a protective effect. In line with our findings, Ahmed et al. also identified MELD score as a predictor of mortality in patients with variceal bleeding, along with Child–Pugh score, ALBI grade, and PALBI grade [28].

This study has several limitations that should be acknowledged. First, its retrospective, single-center design may introduce residual confounding and limit the generalizability of the findings to other clinical settings. Differences in patient populations, management strategies, and endoscopic practices across centers may influence outcomes and therefore restrict the external applicability of our results. Prospective multicenter studies are needed to externally validate these findings. Second, given the relatively small number of rebleeding events, the high predictive performance of the model (AUC 0.981) should be interpreted with caution, as a degree of overfitting cannot be entirely excluded. Although internal validation using bootstrap resampling supported the stability of the main predictors, external validation in larger independent cohorts is required. Third, endoscopic procedures and ultrasound assessments were performed by multiple operators, which may have introduced a degree of inter-observer variability. Fourth, hepatic venous pressure gradient measurements were not available, and liver disease severity was therefore assessed using validated surrogate scores, such as MELD and ALBI, which may not fully capture portal hemodynamic status. In addition, the relatively small number of post-banding ulcer rebleeding events may have limited the statistical power of subgroup analyses.

By separately analyzing variceal and post-banding ulcer rebleeding, this study provides additional insight into the heterogeneous mechanisms underlying early rebleeding after therapeutic EVL and highlights the potential value of tailored risk stratification strategies in patients with cirrhosis.

## 5. Conclusions

Early rebleeding after EVL represents a frequent and severe complication in patients with liver cirrhosis, with a significant impact on short-term outcomes. In our study, hemoglobin level and the number of bands applied during the index procedure emerged as robust independent predictors of early variceal rebleeding, while ALBI grade may represent a potential marker of early variceal rebleeding risk, although larger studies are required to validate its predictive role. Fibrinogen was the only predictor of early PBU rebleeding in multivariable analysis. Moreover, MELD score, serum albumin level, platelet count, and the occurrence of PBU rebleeding independently predicted six-week mortality.

These findings underscore the heterogeneous mechanisms underlying early rebleeding after EVL and highlight the importance of a comprehensive clinical, laboratory, and endoscopic evaluation for risk stratification in cirrhotic patients.

## Figures and Tables

**Table 1:** Baseline clinical, laboratory, and ultrasound characteristics according to rebleeding status.

Variable			No Rebleeding (n = 179)	Variceal Rebleeding (n = 27)	PBU Rebleeding (n = 11)
Gender	Male	n (%)	130 (72.6%)	19 (70.4%)	5 (45.5%)
	Female	n (%)	49 (27.4%)	8 (29.6%)	6 (54.5%)
Age (years)		Mean ± SD	58.9 ± 12.2	55.9 ± 14.3	64.4 ± 12.6
Etiology of cirrhosis	Alcohol-related	n (%)	123 (68.7%)	16 (59.3%)	6 (54.5%)
	Viral-related	n (%)	32 (17.9%)	5 (18.5%)	3 (27.3%)
	Alcohol- and viral-related	n (%)	21 (11.7%)	4 (14.8%)	1 (9.1%)
	Other	n (%)	4 (2.2%)	2 (7.4%)	1 (9.1%)
HCC		n (%)	10 (5.6%)	14 (51.9%)	3 (27.3%)
Immunotherapy		n (%)	1 (0.6%)	5 (18.5%)	0
HE		n (%)	140 (78.2%)	23 (85.2%)	9 (81.8%)
SBP		n (%)	10 (5.6%)	11 (40.7%)	3 (27.3%)
ACLF		n (%)	11 (6.1%)	3 (11.1%)	2 (18.2%)
AKI		n (%)	32 (17.9%)	5 (18.5%)	2 (18.2%)
Clinical presentation	Hematemesis	n (%)	71 (39.7%)	14 (51.9%)	7 (63.6%)
	Melena	n (%)	40 (22.3%)	1 (3.7%)	1 (9.1%)
	Both	n (%)	68 (38%)	12 (44.4%)	3 (27.3%)
Platelets (×10^3^/µL)		Mean ± SD	100.4 ± 69.6	129.2 ± 72.1	106.3 ± 49
Albumin (g/dL)		Mean ± SD	2.8 ± 0.5	2.6 ± 0.7	2.8 ± 0.3
Bilirubin (mg/dL)		Mean ± SD	2.8 ± 3.1	4.5 ± 5.2	4.6 ± 6.5
Fibrinogen (mg/dL)		Mean ± SD	215.5 ± 26.7	191.1 ± 19.5	184.5 ± 11.3
INR		Mean ± SD	1.8 ± 1.3	1.6 ± 0.4	1.8 ± 1.1
Prothrombin time (PT)		Mean ± SD	18.6 ± 5	19.3 ± 3.7	20.8 ± 13
Hemoglobin (g/dL)		Mean ± SD	7.7 ± 1.1	6.0 ± 1.0	6.4 ± 0.8
Rifaximin use		n (%)	143 (79.9%)	11 (40.7%)	5 (45.5%)
Ascites	None/mild	n (%)	87 (48.6%)	8 (29.6%)	4 (36.4%)
	Moderate/severe	n (%)	93 (52%)	19 (70.4%)	7 (63.6%)
Portal vein (PV)	Diameter of PV (mm)	Mean ± SD	13.9 ± 1.6	14.4 ± 1.2	14.3 ± 0.8
	PV ≥ 14 mm	n (%)	103 (57.5%)	22 (81.5%)	9 (81.8%)
	PVT	n (%)	9 (5%)	10 (37%)	1 (9.1%)
Spleen diameter		Mean ± SD	141.3 ± 21.7	145.1 ± 27.1	143.8 ± 29.3
Child–Pugh class	A	n (%)	3 (1.7%)	2 (7.4%)	1 (9.1%)
	B	n (%)	106 (59.2%)	8 (29.6%)	4 (36.4%)
	C	n (%)	71 (39.7%)	17 (63%)	6 (54.5%)
MELD score		Mean ± SD	16.7 ± 5.6	20.4 ± 4.5	17.8 ± 6.8
ALBI grade	1	n (%)	40 (22.3%)	0	0
	2	n (%)	92 (51.4%)	12 (44.4%)	2 (18.2%)
	3	n (%)	47 (26.3%)	15 (55.6%)	9 (81.8%)

ACLF, acute-on-chronic liver failure; AKI, acute kidney injury; ALBI, albumin–bilirubin; HCC, hepatocellular carcinoma; HE, hepatic encephalopathy; INR, international normalized ratio; MELD, Model for End-Stage Liver Disease; PBU, post-banding ulcer; PVT, portal vein thrombosis; SBP, spontaneous bacterial peritonitis; SD, standard deviation.

**Table 2:** Endoscopic characteristics of the study population.

Variable			Without Early Rebleeding (n = 179)	With Early Rebleeding—EV (n = 27)	With Early Rebleeding—PBU (n = 11)
EV grade	F2	n (%)	50 (27.9%)	3 (11.1%)	1 (9.1%)
	F3	n (%)	129 (72.1%)	24 (88.9%)	10 (90.9%)
Cherry spots		n (%)	118 (65.9%)	23 (85.2%)	6 (54.5%)
Active bleeding		n (%)	62 (34.6%)	13 (48.1%)	5 (45.5%)
Gastric varices		n (%)	38 (21.2%)	19 (70.4%)	7 (63.6%)
Portal hypertensive Gastropathy		n (%)	59 (33%)	17 (63%)	5 (45.5%)
Number of rubber bands		Mean ± SD	5.00 ± 1.7	4.3 ± 1.5	6.8 ± 0.4
Consolidation banding sessions		n (%)	103 (57.5%)	6 (22.2%)	3 (27.3%)

EV, esophageal varices; PBU, post-banding ulcer; SD, standard deviation.

**Table 3:** Comparison of continuous variables according to rebleeding status.

Variable	Status	N	Mean	Standard Deviation
Age *p* = 0.162	No rebleeding	179	58.9	12.2
	Variceal rebleeding	27	55.9	14.3
	PBU rebleeding	11	64.4	12.6
	Total	217	58.8	12.5
Albumin *p* = 0.557	No rebleeding	179	2.8	0.5
	Variceal rebleeding	27	2.6	0.7
	PBU rebleeding	11	2.8	0.3
	Total	217	2.7	0.5
Hemoglobin ***p* < 0.001**	No rebleeding	179	7.7	1.1
	Variceal rebleeding	27	6	1
	PBU rebleeding	11	6.4	0.8
	Total	217	7.4	1.3
Total bilirubin ***p* = 0.032**	No rebleeding	179	2.8	3.1
	Variceal rebleeding	27	4.5	5.2
	PBU rebleeding	11	4.6	6.5
	Total	217	3.1	3.7
Fibrinogen ***p* < 0.001**	No rebleeding	179	215.5	26.7
	Variceal rebleeding	27	191.1	19.5
	PBU rebleeding	11	184.5	11.3
	Total	217	210.9	27.3
Platelets *p* = 0.132	No rebleeding	179	100.4	69.6
	Variceal rebleeding	27	129.2	72.1
	PBU rebleeding	11	106.3	49
	Total	217	104.2	69.4
INR *p* = 0.666	No rebleeding	179	1.8	1.3
	Variceal rebleeding	27	1.6	0.4
	PBU rebleeding	11	1.8	1.1
	Total	217	1.75	1.2
Prothrombin time (PT) *p* = 0.394	No rebleeding	179	18.6	5
	Variceal rebleeding	27	19.3	3.7
	PBU rebleeding	11	20.8	13
	Total	217	18.8	5.5
Portal vein diameter *p* = 0.300	No rebleeding	179	13.9	1.6
	Variceal rebleeding	27	14.4	1.2
	PBU rebleeding	11	14.3	0.8
	Total	217	14	1.5
Spleen diameter *p* = 0.691	No rebleeding	179	141.3	21.7
	Variceal rebleeding	27	145.1	27.1
	PBU rebleeding	11	143.8	29.3
	Total	217	141.9	22.7
Number of bands ***p* = 0.001**	No rebleeding	179	5	1.7
	Variceal rebleeding	27	4.3	1.5
	PBU rebleeding	11	6.8	0.4
	Total	217	5	1.8
Child–Pugh score *p* = 0.706	No rebleeding	179	9.3	2.1
	Variceal rebleeding	27	9.2	2.1
	PBU rebleeding	11	9.8	2.3
	Total	217	9.3	2.1
MELD score ***p* = 0.006**	No rebleeding	179	16.7	5.6
	Variceal rebleeding	27	20.4	4.5
	PBU rebleeding	11	17.8	6.8
	Total	217	17.2	5.6

INR, international normalized ratio; PT, prothrombin time; MELD, Model for End-Stage Liver Disease; PBU, post-banding ulcer. Bold values indicate statistically significant results (*p* < 0.05).

**Table 4:** One-way ANOVA and post hoc comparisons for variables showing significant differences according to rebleeding status.

Variable	F (df)	*p*-Value (ANOVA)	Post hoc Comparisons (LSD)
Total bilirubin	F (2, 214) = 3.499	0.032	No rebleeding vs. EV: *p* = 0.025
			No rebleeding vs. PBU: *p* = 0.115
			EV vs. PBU: *p* = 0.941
Fibrinogen	F (2, 214) = 16.996	**<0.001**	No rebleeding vs. EV: *p* < 0.001
			No rebleeding vs. Ulcers: *p* < 0.001
			EV vs. PBU: *p* = 0.472
Hemoglobin	F (2, 214) = 33.195	**<0.001**	No rebleeding vs. EV: *p* < 0.001
			No rebleeding vs. PBU: *p* < 0.001
			EV vs. PBU: *p* = 0.214
Number of bands	F (2, 214) = 27.813	**<0.001**	No rebleeding vs. EV: *p* = 0.028
			No rebleeding vs. PBU: *p* < 0.001
			EV vs. PBU: *p* < 0.001
MELD score	F (2, 214) = 5.324	**0.006**	No rebleeding vs. EV: *p* = 0.001
			No rebleeding vs. PBU: *p* = 0.538
			EV vs. PBU: *p* = 0.182

F: statistic from one-way ANOVA; df: degrees of freedom; EV, esophageal varices; PBU, post-banding ulcer; MELD, Model for End-Stage Liver Disease. Bold values indicate statistically significant results (*p* < 0.05).

**Table 5:** Variables significantly associated with early variceal rebleeding.

Variable	χ^2^	*p*-Value	Cramér’s V
SBP	31.670	<0.001	0.392
HCC	48.788	<0.001	0.487
Immunotherapy	26.761	<0.001	0.360
Rifaximin	19.053	<0.001	0.304
Ascites	0.696	0.404	–
PVT	28.710	<0.001	0.373
EV size	9.887	0.002	0.219
Cherry spots	4.031	0.045	0.140
Portal hypertensive Gastropathy	11.634	0.001	0.238
Gastric varices	33.432	<0.001	0.403
ALBI grade	11.092	0.004	0.232

Degrees of freedom vary according to variable categories. χ^2^ = chi-square statistic; Cramér’s V = effect size. Only variables with statistically significant associations (*p* < 0.05) are shown. ALBI, albumin–bilirubin; EV, esophageal varices; HCC, hepatocellular carcinoma; PVT, portal vein thrombosis; SBP, spontaneous bacterial peritonitis.

**Table 6:** Variables significantly associated with post-banding ulcer rebleeding.

Variable	χ^2^	*p*-Value	Cramér’s V
Rifaximin use	7.136	0.008	0.194
SBP	7.646	0.006	0.201
HCC	7.646	0.006	0.201
Esophageal varices size	6.001	0.014	0.178
Gastric varices	10.751	0.001	0.238
ALBI grade	15.175	0.001	0.283

Degrees of freedom vary according to variable categories. χ^2^ = chi-square statistic; Cramér’s V = effect size. Only variables with statistically significant associations (*p* < 0.05) are shown. ALBI, albumin–bilirubin; SBP, spontaneous bacterial peritonitis.

## Data Availability

Supporting data are available upon request from the corresponding author.

## Abbreviations

The following abbreviations are used in this manuscript:

EVL	endoscopic variceal ligation
PBU	post-banding ulcer
MELD	model for end stage liver disease
VB	variceal bleeding
NSBBs	nonselective beta-blockers
HVPG	hepatic venous pressure gradient
ESGE	European Society of Gastrointestinal Endoscopy
PPI	proton pump inhibitors
UGE	upper gastrointestinal endoscopy
ACLF	acute-on-chronic liver failure
AKI	acute kidney injury
ALBI	albumin–bilirubin
HCC	hepatocellular carcinoma
HE	hepatic encephalopathy
INR	international normalized ratio
PVT	portal vein thrombosis
SBP	spontaneous bacterial peritonitis
SD	standard deviation
EV	esophageal varices
PT	prothrombin time
Df	degrees of freedom
N	number of patients in full cohort
N	number of patients in subcohort
AUC	area under the curve
ANOVA	one-way analysis of variance
LSD	least significant difference
KM	Kaplan–Meier
BCa	generating bias-corrected and accelerated
VIF	Variance Inflation Factor
